# Autophagy in striated muscle diseases

**DOI:** 10.3389/fcvm.2022.1000067

**Published:** 2022-10-13

**Authors:** Haiwen Li, Lingqiang Zhang, Lei Zhang, Renzhi Han

**Affiliations:** ^1^Department of Surgery, Davis Heart and Lung Research Institute, Biomedical Sciences Graduate Program, Biophysics Graduate Program, The Ohio State University Wexner Medical Center, Columbus, OH, United States; ^2^State Key Laboratory of Proteomics, National Center of Protein Sciences (Beijing), Beijing Institute of Lifeomics, Beijing, China; ^3^Department of Anatomy and Neurobiology, Shanghai Yangzhi Rehabilitation Hospital, Shanghai Sunshine Rehabilitation Center, School of Medicine, Tongji University, Shanghai, China

**Keywords:** autophagy, mitophagy, muscular dystrophy, cardiomyopathy, myopathy, gene therapy, heart disease, skeletal muscle disease

## Abstract

Impaired biomolecules and cellular organelles are gradually built up during the development and aging of organisms, and this deteriorating process is expedited under stress conditions. As a major lysosome-mediated catabolic process, autophagy has evolved to eradicate these damaged cellular components and recycle nutrients to restore cellular homeostasis and fitness. The autophagic activities are altered under various disease conditions such as ischemia-reperfusion cardiac injury, sarcopenia, and genetic myopathies, which impact multiple cellular processes related to cellular growth and survival in cardiac and skeletal muscles. Thus, autophagy has been the focus for therapeutic development to treat these muscle diseases. To develop the specific and effective interventions targeting autophagy, it is essential to understand the molecular mechanisms by which autophagy is altered in heart and skeletal muscle disorders. Herein, we summarize how autophagy alterations are linked to cardiac and skeletal muscle defects and how these alterations occur. We further discuss potential pharmacological and genetic interventions to regulate autophagy activities and their applications in cardiac and skeletal muscle diseases.

## Introduction

Autophagy is an evolutionarily conserved, catabolic process that digests undesirable cytoplasmic components and organelles in the lysosomes, allowing the cell to reuse the materials and maintain cellular homeostasis. Numerous studies have demonstrated the crucial roles of autophagy in many biological processes, such as development, aging, and immune responses (1–5). Emerging evidence has linked aberrant autophagy execution to many human diseases, such as cardiomyopathies and muscular dystrophies (1–5).

**FIGURE 1:**
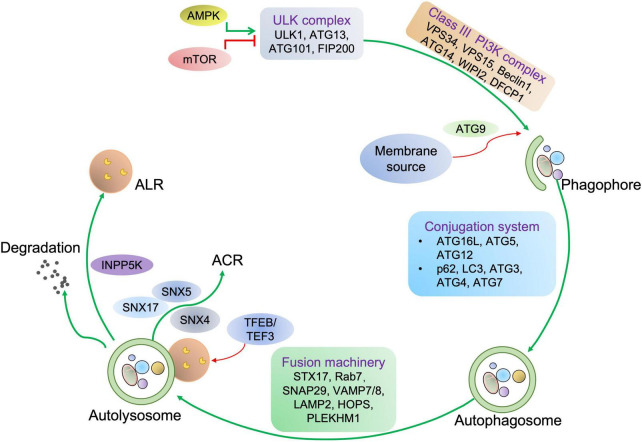
Autophagy process in mammalian cells. For autophagy initiation, environmental factors and metabolic stimuli are sensed by AMPK or mTORC1. The autophagic cargo undergoes a serial of processes, including the phagophore and autophagosome formation, autolysosome development before the cargo is degraded, concomitant with lysosome and autophagosome recycling. Once autophagy is induced, ULK1 is activated and then associated with the class III PI3K complex, eliciting the production of PI3P on the phagophore membrane, which further recruits WD repeat domain phosphoinositide-interacting protein 2 (WIPI2) and double FYVE domain-containing protein 1 (DFCP1). Next, the ATG12-ATG5-ATG16L1 complex binding to WIPI2 is responsible for providing membranes from other organelles, including the plasma membrane, mitochondria, recycling endosomes, and Golgi complex. Then, ATG9 drives membrane expansion by delivering phospholipids. Moreover, the ATG12-ATG5-ATG16L1 complex promotes the conjugation of LC3, in which ATG4 cleaves LC3 to generate LC3-I, which further covalently bonds with phosphatidylethanolamine for LC3-II formation. LC3-II is a specific marker of autophagy where autophagosome-residing LC3-II can specifically associate with autophagy receptors with LC3-interacting motifs like p62. Finally, autophagosome-lysosome fusion is commonly mediated by UV radiation resistance-associated gene protein (UVRAG), homotypic fusion and protein sorting (HOPS), syntaxin-17 (STX17), vesicle-associated membrane protein 7/8 (VAMP7/8), synaptosome associated protein 29 (SNAP29), PLEKHM1, and the trafficking protein Rab-7. Concomitant with cargo removal, autophagosome effector proteins are either degraded or recycled by the Sorting Nexin 4/5/17 (SNX4/5/17) complex, and lysosomes maybe undergo the INPP5K-mediated ALR process.

**FIGURE 2:**
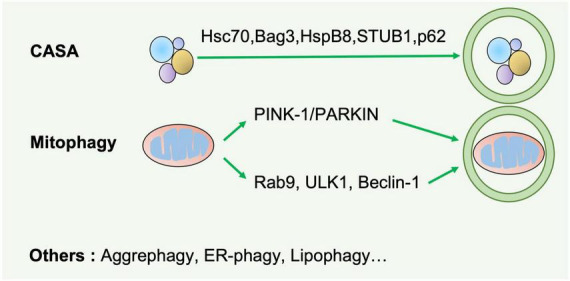
Selective autophagy. Selective autophagy delivers specific cargos for degradation and recycling, such as protein aggregates, mitochondria, and endoplasmic reticulum. The recognition of these cargoes requires selective autophagy receptors specifically binding LC3-II of autophagosomes and are thereby removed by lysosomes. CASA is a selective tension-induced autophagy pathway mediated by BAG3. Moreover, mitophagy is divided into two branches: the classical PINK1/PRAK2-mediated process and alternative mitophagy mediated by Rab9. Other types of selective autophagy include aggrephagy, ER-phagy, lipophagy et al.

Based on the cargo sequestration methods, autophagy can be classified into three primary types: microautophagy, macroautophagy, and chaperone-mediated autophagy. Macroautophagy (henceforth termed autophagy) is well characterized among these types. Cells can sequester cytosolic materials into double-membrane vesicles (known as autophagosomes), and degrade these cargos by fusing with lysosomes during this process (6) (Figure 1). Based on the cargos, autophagy can be separated into bulk autophagy and selective autophagy such as ER-phagy, aggrephagy (7), and PINK1 (PTEN-induced kinase 1)/PRAK2 (parkin RBR E3 ubiquitin protein ligase)-mediated mitophagy (8) (Figure 2). This review mainly focuses on bulk autophagy and mitophagy in striated muscle diseases.

**FIGURE 3:**
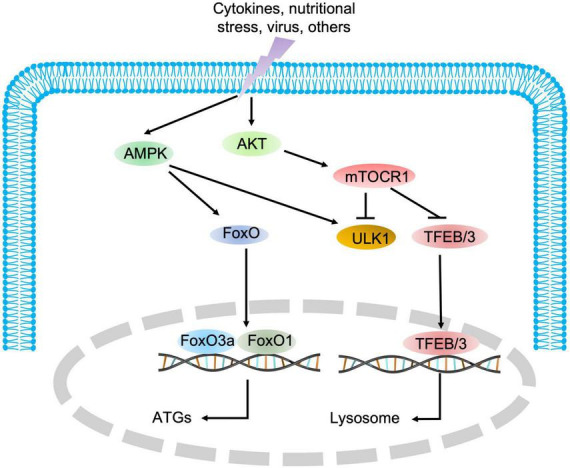
The regulatory signaling pathways of autophagy. AMPK and mTOR are the major positive and negative regulatory signaling pathways of autophagy, respectively. Once AMPK is activated, the downstream substrate ULK1 is phosphorylated, and FoxO transcription factors are translocated into the nucleus to promote the expression of autophagic genes. Conversely, mTOR blocks autophagic initiation by negatively phosphorylating ULK1 and inhibits lysosome biosynthesis by blocking TFEB/TFE3 translocation into the nucleus.

As indicated in Figure 1, autophagy is a multiphasic process that involves the sequential and selective recruitment of autophagy-related (ATG) proteins. The complex process includes initiation/nucleation, phagophore formation, autophagosome formation, autophagosome-lysosome fusion, cargo degradation, and autophagic lysosome reformation (ALR) or emerging autophagosomal components recycling (ACR) (9). Different ATG proteins or complexes are involved in these steps. As shown in Figure 3, key upstream regulators of this process include the major inhibitor mammalian target of rapamycin (mTOR) and the primary activator AMP-activated kinase (AMPK). The main downstream phosphorylation substrates of AMPK are Unc-51-like kinase (ULK1) (10) and Forkhead box protein O (FoxO) (11, 12), in which the former is a crucial initiator of autophagy and the latter regulates the transcription of genes related to autophagy. Moreover, mTOR, particularly mTOCR1, suppresses autophagy through phosphorylating ULK1 at different sites (10) and transcription factor EB (TFEB)/transcription factor E3 (TFE3), two key proteins of lysosome biosynthesis (13, 14). The details of the autophagy process have been well reviewed in other studies (4, 15).

The role of autophagy in various pathophysiological processes has spurred great efforts toward identifying clinically druggable autophagic targets to prevent or cure human diseases, including cardiac and skeletal myopathies. Here, we systematically summarize the current insights into the role of autophagy in human diseases related to striated muscle and therapeutic strategies in preclinical development.

## Aberrant autophagy in heart diseases

Heart disease is the leading cause of morbidity and death worldwide (16). Adult cardiomyocytes, the essential cellular component of cardiovascular system, are mostly long-lived and rarely renewed, implying that these cells heavily rely on intact autophagy to remove impaired proteins and organelles during their long life (5). Aberrant autophagy can lead to various heart defects.

### Bulk autophagy in heart diseases

As illustrated by the genetic models of several essential or ancillary genes related to autophagy (Table 1), autophagy aberration predisposes the organisms to develop heart disorders under either basal or stress conditions (1, 17). For instance, three different cardiomyocyte-specific ATG5 conditional knockout (KO) mouse models display left ventricular dilatation and cardiac dysfunction without or with pressure overload (18, 19). Vacuolar protein sorting 34 (Vps34) negatively correlates with human hypertrophic cardiomyopathy (HCM) characterized by thickening of the heart muscle, in consistence with the observation that disruption of *Vps34* causes cardiac hypertrophy in mice by accumulating ubiquitinated Crystallin Alpha B (CryAB) (20). Muscle-specific conditional KO of ATG14 causes early death and HCM with abnormal accumulation of autophagic cargoes in heart (21). Moreover, other core autophagy factors such as Beclin-1 (22), mTORC1 (23–25), and PLEKHM2 (Pleckstrin Homology and RUN Domain Containing M2) (26) are also essential for cardiac homeostasis, and their ectopic activity can cause heart defects.

**TABLE 1:** Autophagy in heart diseases.

Disease	Target	Model	Main conclusions	References
Sepsis	Beclin-1	WT, *Becn*^+/–^ and TG mice	Beclin-1 promotes autophagy, suppresses mTOR signaling, improves cardiac function, and alleviates inflammation and fibrosis	(22)
HF	AMPKα2	*Ampk*α*2* KO mice	AMPKα2^–/–^ mice exhibits an exacerbation of early TAC-induced HF by suppressing cardiac mitophagy	(62)
HF	ATG5	*Atg5* cKO mice	The defect in cardiac Atg5-dependent autophagy reduces mitochondrial number and alters subcellular Ca^2+^ cycling	(60)
AVSD	TAMM41	*Tamm41* KO zebrafish	TAMM41 deficient causes heart valve abnormalities by impairing PINK1-PARK2 dependent mitophagy	(63)
CM	LAMP2	*Lamp2* KO mice, patients	Heart contractility is severely reduced along with accumulation of autophagic material in striated myocytes	(32, 33)
CM	LAMP2	*Lamp2*-KO iPSC-CMs	Impaired fusion of lysosomes with autophagosomes in *Lamp2*-KO iPSC-CMs	(34)
CM	ATG5	*Atg5*-cKO mice	Loss of Atg5 causes cardiac hypertrophy, left ventricular dilatation, and contractile dysfunction	(18)
CM	ATG5	*Atg5*-cKO mice	Loss of Atg5 increases in left ventricular dimension and decrease in fractional shortening	(19)
HF	PSMB8	*Psmb8* KO mice	Loss of PSMB8 attenuates pressure overload–induced cardiac hypertrophy	(28)
DC	Nrf2	*Nrf2* KO, *Nrf2* TG, *Atg5* cKO mice	Loss of ATG5 causes early onset and accelerated development of cardiomyopathy in T1D, and Nrf2 deficient can rescue these adverse phenotypes	(29)
HF	GRK4	*Grk4* ^A486V^ TG mice, *Grk4*-cKO mice	GRK4 enhances MI-induced cardiac injury by decreasing Beclin-1 expression, repressing autophagy, and enhancing apoptosis	(30)
Hypertrophy	Vps34	*Vps34*-cKO mice	Vps34-cKO mice develop cardiomyopathy by suppressing autophagy	(20)
HF	miR-212/132	*miR-212/132* TG and KO mice	Both miR-212 and miR-132 leads to hyperactivation of pro-hypertrophic calcineurin/NFAT signaling by FoxO3 and an impaired autophagic response	(43)
Hypertrophy	miR-199a	*miR-199a* TG mice	miR-199a impairs cardiomyocyte autophagy by regulating GSK3β/mTOR signaling	(44)
CM	BAG3	*hBag3^*P*209*L*^-eGFP* mice	hBAG3^P209L^ leads to sarcomere disruption by sequestering autophagy machinery	(48)
HF	BAG3	*Bag3*-cKO mice	BAG3 haploinsufficient mice display reduced maximum force-generating capacity and increased myofilament ubiquitination	(50)
Hypertrophy	TSC2	*Tsc2*-cKO mice	TSC2^– /–^ mice show cardiac dysfunction and cardiomyocyte hypertrophy by inhibiting autophagic flux	(23)
Hypertrophy	TSC2	*Tsc2*^S1365A^ KI, *Tsc2*^S1365E^ KI mice	TSC2^S1365A^ KI mice develop worse heart disease and have higher mortality after sustained pressure overload of the heart, owing to mTORC1 hyperactivity	(24)
HCM	PKG1α	*Pkg1*α^C42S^, *Tsc2*^S1365A^ KI mice	Oxidation of PKG1α at C42 results in amplified PO-stimulated mTORC1 activity and cardiac hypertrophy	(25)
MI	LAPTM4B	*Laptm4b* KO mice	LAPTM4B^– /–^ mice has a significantly increased infarct size	(27)
Atrophy	Thbs1	*Thbs1* TG mice, *Thbs1* KO mice	*Thbs1* TG mice display lethal cardiac atrophy *via* activating PERK-eIF2α-ATF4-mediated autophagy, *Thbs1*^– /–^ mice develop cardiac hypertrophy	(39)
CM	RagA/B	*RagA/B*-cKO mice	RagA/B-cKO mice exhibits enlargement of the LV and contractile dysfunction	(38)
DCM	PLEKHM2	Patients	PLEKHM2 mutation causes aberrant localization of lysosomes and defective autophagy flux	(26)
HF	MiR-221	*miR-221* TG mice	miR-221 induces HF by activating mTOR and inhibiting autophagy	(42)
HCM	ATG14	*Atg14*-cKO mice	Atg14 deficient causes abnormal accumulation of autophagic cargoes in heart	(21)
HF	Kansl1	*Kansl1*^+/–^ mice	Kansl1 insufficiency results in defective cardiac functions	(31)
HCM	LncRNA Gm15834	TAC, Ang-II mice model	Gm15834 enhances autophagic activity and promotes myocardial hypertrophy	(45)
DC	Rab9	*Ulk1*-cKO mice, *Rab9*^S179A^ KI mice	Ulk1-Rab9-dependent alternative mitophagy and upregulation of TFE3 safeguards the heart against obesity cardiomyopathy	(71)
I/R injury	Rab9	*Atg7*-cKO, *Ulk1*-cKO, *Park2* KO, *Rab9*-KI mice	Ulk1/Rab9/Rip1/Drp1 pathway protected the heart against ischemia damage by activating autophagy	(70)
DC	ACC2	*Acc2* KO mice	Increasing cardiac FAO protects against cardiomyopathy in chronically obese mice	(64)
HF	p53	*p53*-*Park2* dKO mice	Cytosolic p53 impairs mitophagy and facilitates mitochondrial dysfunction and heart failure in mice	(65)
MI	PARK2	*Park2* KO mice	KO mice reduces survival and develops larger infarcts after MI	(53)
HCM	PINK1	*Pink1* KO mice	KO mice develop left ventricular dysfunction and cardiac hypertrophy through the impairment of mitochondrial function and the increase in ROS	(55)
DCM	BNIP3	*Bnip3* KO, *Nix*-cKO mice	Bnip3 and Nix is sufficient for cardiomyopathy development and essential for cardiac remodeling	(56)
DCM	Mfn2	*Mfn2* cKO mice	Mfn2 deficiency causes dilated cardiomyopathy due to the suppression of mitophagy	(57)
MI	Mfn1/2	*Mfn1/2* dKO mice	dKO mice are protected against acute MI due to impaired mitochondria/SR tethering	(58)
CM	DMD	*mdx* mice	The defect in PINK1/PRKN-mediated mitophagy contributes to dystrophic cardiomyopathy	(108)
DC	ATG7, PARK2	*Atg7* cKO mice, *Park2* KO mice	Atg7-dependent mitophagy protects against hypertrophy and diastolic dysfunction	(61)
MI	RhoA	WT mice	RhoA protects MI through activating PINK1/PRKN-mediated mitophagy	(66)
DCM	SDHAF4	*Sdhaf4*-cKO mice	SDHAF4 deficient impairs complex II assembly and activates mitophagy, thereby causing progressive DCM	(67)

CM, cardiomyopathy; HF, heart failure; HCM, hypertrophic cardiomyopathy; MI, myocardial infarction; DCM, dilated cardiomyopathy; DC, diabetic cardiomyopathy; WT, wild-type; KO, knock-out; KI, knock-in; TG, transgenic; cKO, conditional knock-out; dKO, double knock-out; T1D, type 1 diabetes; iPSC-CMs, human induced pluripotent stem cell-derived cardiomyocytes; AVSD, sporadic atrioventricular septal defect; LV, left ventricle; Ang-II model, angiotensin-II-induced cardiac hypertrophy model; TAC, transverse aortic constriction; I/R injury, ischemia-reperfusion injury.

A large body of evidence has shown that alterations in regulatory proteins related to autophagy compromise cardiac function by modulating the core autophagy machinery. For example, mice with a disruption in lysosomal-associated transmembrane protein 4B (LAPTM4B) are susceptible to ischemia-reperfusion (I/R) injury by repressing mTORC1-mediated TFEB transcription (27). Upregulation of immunoproteasome catalytic subunit β5i leads to cardiac hypertrophy and heart failure (HF) by promoting ATG5 degradation (28), while Nrf2 ablation slows the progression of diabetic cardiomyopathy (DC) in cardiomyocyte-specific ATG5 KO mice (29). G protein-coupled receptor kinase 4 (GRK4) aggravates cardiomyocyte injury during myocardial infarction (MI) by inhibiting histone deacetylase 4 (HDAC4)-mediated Beclin-1 transcription, while MI-induced cardiac dysfunction and remodeling are improved by deleting cardiomyocyte-specific GRK4 (30). Moreover, other regulatory factors of autophagy, including KAT8 Regulatory NSL complex subunit 1 (KANSL1) (31), Lysosome-associated membrane protein 2 (LAMP2) (32–34), insulin-like growth factor 1 receptor (IGF1R) (35) and HDAC (36, 37), also play imperative roles in maintaining cardiac fitness, and their abnormality leads to heart diseases. These findings demonstrate that autophagy is important for cardiac function. However, in some cases, overactivation of autophagy can compromise cardiac fitness. For example, cardiac-specific knockout of the genes encoding the lysosomal proteins Rag family protein A/B (RagA/B) causes lysosomal storage disorder characterized by increased autophagosome accumulation due to the activation of yes-associated protein 1 (YAP1)-TFEB transcription (38). Furthermore, cardiomyocyte-specific transgenic thrombospondin-1 (Thbs1) mice develop lethal cardiac atrophy due to overactivation of PERK/ATF4-mediated autophagy (39).

MicroRNAs (miRNAs) and long non-coding RNAs (lncRNAs) can modulate the expression of autophagy-related proteins and pathways (40) and are potential druggable targets for heart disease treatment (41). miR-221 induces HF by inhibiting mTOR-mediated autophagy, while rapamycin treatment abolishes the miR-221-induced suppression of autophagy and cardiac remodeling (42). The defective autophagic response and HF are caused when FoxO3 is inhibited by cardiomyocyte-specific overexpression of miR-212/132 (43) or mTORC1 is activated by miR-199a (44). Moreover, the suppression of lncRNA Gm15834 mitigates autophagy-mediated myocardial hypertrophy by downregulating ULK1 in mice (45).

### Chaperone-assisted selective autophagy in heart diseases

The chaperone-assisted selective autophagy (CASA) machinery consists of the chaperones heat shock protein 70 (HSC70), heat shock protein beta-8 (HSPB8), co-chaperone Bcl2-associated athanogene 3 (BAG3), STIP1 homology and U-Box containing protein 1 (STUB1), and autophagic receptor sequestosome-1 (SQSTM1, also known as p62). CASA primarily mediates the autophagic degradation of filamin C, which is involved in actin–actin and actin–integrin interactions in muscle tissues (46, 47). Emerging evidence has demonstrated that BAG3 plays an essential role in maintaining cardiac function (46, 47). Human BAG3^*P*209*L*^-eGFP expression in mice causes the disintegration of Z-disc, accumulation of protein aggregates and development of early-onset restrictive cardiomyopathy with increased mortality, in line with the observation in BAG3^*P*209*L*^ patients (48, 49). Histological and biochemical assays revealed the alterations in protein quality control system and autophagy in heart tissues from BAG3^*P*209*L*^-eGFP transgenic mice and patients (48, 49). Similarly, compromised CASA impairs cardiomyocyte contractility and leads to HF in BAG3 heterozygous KO mice (50). A recent study showed that loss-of-function of BAG5 (one of the BAG3 paralogs) also led to dilated cardiomyopathy (DCM), which is characterized by enlargement and dilation of the ventricles along with impaired contractility, in mice and humans partly by disrupting the interaction with HSC70 (51).

### Mitophagy in heart diseases

Defects in mitophagy, a selective autophagy targeting mitochondria, have been closely linked to cardiac disorders (1). The classic PINK1/PRAK2-mediated mitophagy is essential for cardiac mitochondrial fitness and protects the heart from cardiomyopathy (52). Once mitochondria are damaged, PINK1 is increased and activated by autophosphorylation on the outer mitochondrial membrane (OMM). Activated PINK1 further phosphorylates ubiquitin, promoting the ubiquitin E3 ligase PRAK2 recruitment to mitochondria. Meanwhile, phospho-ubiquitin recruits and binds with autophagy receptors to initiate autophagosome formation. Parkin functions as an amplifier of mitophagy through further ubiquitination of mitochondrial proteins.

*Park2* global knockout mice display a decrease in survival and develop larger infarcts than wild-type (WT) mice after MI (53), and cardiomyocyte-specific deletion of *Park2* manifests cardiac hypertrophy at birth and early lethality (54). Systematic knockout of *Pink1* leads to left ventricular defects and age-dependent cardiac hypertrophy by compromising mitochondrial fitness and increasing oxidative stress (55). Additionally, heart defects are also observed in the mouse models related to other key mitophagy factors, such as double KO of Bcl2 interacting protein 3 (BNIP3) and Bcl2 interacting protein 3 (Nix/BNIP3L) (56), cardiomyocyte-specific KO of mitophagy receptor Mitofusin 2 (Mfn2) (57), and inducible double KO of cardiac Mfn1/2 (58). As expected, the impairment in classic autophagy machinery including ATG5 (59, 60), ATG7 (61) as well as AMPKα2 (62) causes heart defects by altering mitophagy. In addition to the core components, the maintenance of heart fitness also requires the involvement of some other regulatory proteins of PINK1/PRAK2-mediated mitophagy such as TAM41 Mitochondrial Translocator Assembly and Maintenance Homolog (TAMM41) (63), acetyl-CoA carboxylase 2 (ACC2) (64), tumor protein p53 (p53) (65), Ras homolog family member A (RhoA) (66), and succinate dehydrogenase assembly factor 4 (SDHAF4) (67).

Mitophagy also plays a crucial role in preventing diabetes-induced cardiomyopathy (68), particularly for ULK1/Rab9 (Ras-related protein 9)-mediated mitophagy (69). As an alternative mitophagy, energy stress activates AMPK-mediated phosphorylation of Ulk1. Phosphorylated Ulk1 interacts with and further phosphorylates the Golgi-derived membrane-associated Rab9. Phosphorylated Rab9 forms a complex with receptor interacting protein kinase-1 (Rip1) and dynamin-related Protein 1 (Drp1), thereby catalyzing the phosphorylation of Drp1 by Rip1. Mitochondria with phosphorylated Drp1 are recognized and engulfed by Rab9-associated membranes, and finally degraded by lysosomes. Recent studies showed that Ulk1/Rab9-mediated mitophagy protected the heart against ischemic damage (70) and obesity-associated cardiomyopathy (71) in mice.

## Targeting autophagy for the treatment of heart diseases

**FIGURE 4:**
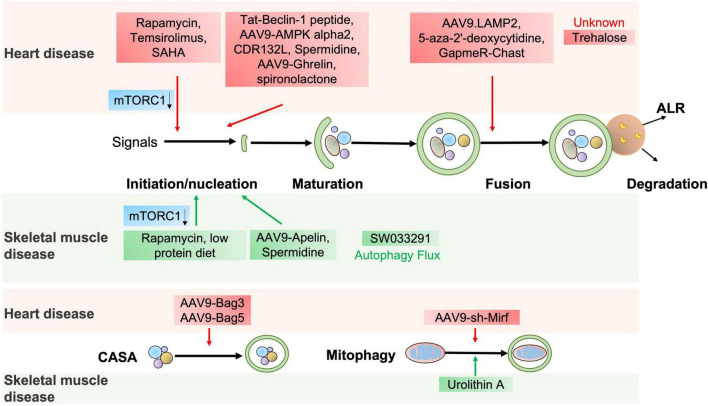
The potential autophagic targets for striated muscle disease treatment. The diagram includes current drug candidates (small molecules, peptides, ASO, and gene therapy) that act at different stages of the autophagy and might be beneficial in heart or skeletal muscle diseases.

The abovementioned evidence indicates that autophagy is essential for cardiac homeostasis and function. Stimulation of autophagy can protect against cardiac defects, as supported by the fact that several autophagy activators manifest a potent therapeutic potential for cardiac disorders (72) (Figure 4 and Table 2).

**TABLE 2:** Targeting autophagy for striated muscle disease treatment.

Disease	Treatment	Model	Main conclusions	References
MI	AAV9-Ghrelin	CD1 mice	Ghrelin markedly reduces infarct size and preserves cardiac function	(79)
I/R injury	rAAV9-BAG3	FVB mice	BAG3 decreases infarct size and improves left ventricular function after I/R	(80)
HF	rAAV9-BAG3	C57/BL6 mice	BAG3 rescues maximum force-generating capacity and CASA protein turnover	(50)
DCM	AAV9-BAG5	*Bag5^*R*197*Ter*^* KI mice	BAG5 can suppress the ventricular arrhythmias with improved left ventricular dilatation and systolic function	(51)
HF	Spermidine	*Dahl* salt-sensitive rats	Spermidine can reduce systemic blood pressure, prevent cardiac hypertrophy and display a decline in diastolic function	(73)
MI	Trehalose	C57/BL6 mice	Trehalose can reduce left ventricular (LV) dilation and increase ventricular function	(74)
AIC	Spironolactone, rapamycin	AIC zebrafish and mice	Spironolactone and rapamycin can reverse the decline in cardiac function and the suppression of autophagic flux in an ATG7-dependent fashion	(75)
CM	Temsirolimus	*Lmna ^*H*222*P/H*222*P*^* mice	Temsirolimus can reactivate autophagy and improve cardiac function by blocking mTORC1 and ERK1/2 activity	(76)
I/R injury	SAHA	Mice and rabbit	SAHA can reduce infarct size and preserve systolic function	(77)
I/R injury	Tat-Beclin-1	WT, *Atg7* KO mice	Tat-Beclin-1 can reduce infarct size and improve contractile function	(82)
Hypertrophy	Rapamycin	*miR-199a* TG mice	Rapamycin can attenuate cardiac hypertrophy by activating autophagy	(44)
Sepsis	Tat-Beclin-1	WT, *Becn*^+/–^ mice	Tat-Beclin-1 can ameliorate cardiac function and survival, attenuate inflammation	(22)
HF	AntimiR-132	TAC mice	AntimiR-132 can prevent pressure-overload-induced heart failure by up-regulating the expression of FoxO3	(43)
HF	AntimiR-132	*miR-212/132* TG mice, MI pig model	AntimiR-132 can ameliorate cardiomyocyte dysfunction, improve HF without safety concerns	(84)
HF	CDR132L	MI pig model	CDR132L (antimiR-132) can improve cardiac function and reverse cardiac remodeling without toxic side effects	(83)
HF	CDR132L	HF patients	CDR132L can induce significant QRS narrowing and show the trend of the decrease in cardiac fibrosis and safety	(85)
HF	GapmeR-Chast	TAC mice	GapmeR-Chast can prevent and improve TAC-induced adverse cardiac remodeling and hypertrophy	(86)
MI	AAV9-sh-Mirf	MI mice	AAV9-sh-Mirf can improve myocardial injury and protect heart function	(87)
HF	AAV9-AMPKα2	TAC mice	AMPK α2 can protect mice against TAC-induced HF through increasing cardiac mitophagy	(62)
DMD	Urolithin A	*Dmd* worm, *mdx*, *mdx/Utr* dKO mice	Urolithin A can enhance skeletal muscle respiratory capacity and improve MuSCs’ regenerative ability by activating mitophagy	(145)
SP	Urolithin A	Aged patients	Urolithin A can promote the expression of skeletal muscle mitochondrial genes	(146)
SP	SW033291	Aged mice	SW033291 can improve aged muscle mass, strength and exercise	(98)
Myopathy	Rapamycin	*Cox15^sm/sm^* mice	Rapamycin can improve exercise, muscle fiber size, and myopathic histology	(126)
UCMD	Spermidine	*Col6a1* KO mice	Spermidine can improve the force contractile and muscle histological defects	(147)
UCMD	Low protein diet	UCMD patients	Low protein diet can reduce fiber apoptosis and improve mitochondrial function	(148)
MM	Rapamycin	Deletor mice and MM patients	Rapamycin can restore mitochondrial recycling	(128)
SP	AAV9-Apelin	Aged mice	AAV9-apelin can enhance muscle functions including exercise, force and increase muscle mass	(99)
Danon	5-Aza-2′-deoxycytidine	iPSC-CMs	5-Aza-2′-deoxycytidine can reactivate LAMP2 and ameliorate autophagy failure	(78)
Danon	AAV9-LAMP2B	*Lamp2* KO mice	AAV9-LAMP2B can improve autophagic flux and cardiac function	(81)

MuSC, muscle stem cell; Cox15^sm/sm^, muscle-specific Cox15 knockout; CM, cardiomyopathy; I/R injury, ischemia-reperfusion injury; AIC, anthracycline-induced cardiotoxicity; TAC, transverse aortic constriction; MI, myocardial infarction; SAHA, suberoylanilide hydroxamic acid; SP, sarcopenia; UCMD, Ullrich congenital muscular dystrophy; MM, mitochondrial myopathy.

The autophagy agonist spermidine, a natural polyamine usually found in mammals, exerts cardioprotective effects including a decrease in cardiac hypertrophy and maintenance of diastolic function in mice and rats (73). Trehalose, a natural non-reducing disaccharide, significantly reduces ischemic remodeling, cardiac dysfunction, and HF in a chronic MI mouse model by activating TFEB-mediated autophagy (74). Anthracycline, including doxorubicin (DOX), is an effective antitumor drug, but the dose-dependent cardiotoxicity limits its application. Recent findings have revealed that anthracycline-induced cardiotoxicity (AIC) was associated with autophagy suppression (75). The Food and Drug Administration (FDA)-approved autophagy activators such as spironolactone, pravastatin, and minoxidil can mitigate AIC by activating ATG7-dependent autophagy (75). Moreover, the beneficial effects of treating autophagy-related heart diseases are also observed with other reagents, like rapamycin for cardiac hypertrophy (44), a rapamycin analog temsirolimus for LMNA-related heart defects (76), an FDA-approved HDAC inhibitor SAHA for MI (77), and a DNA demethylating agent 5-aza-2′-deoxycytidine for the heart defects related with Danon disease (78).

Emerging studies have shown that gene therapy may offer a promising approach for heart disease treatment. AAV9-Ghrelin preserves cardiac function and reduces infarct size after MI, *via* activating autophagy and eradicating damaged mitochondria after MI (79). The overexpression of rAAV9-BAG3 decreases infarct size and improves left ventricular function after I/R injury by activating autophagy and apoptosis (80). Moreover, similar improvements are also observed in AAV9-BAG3 for HF (48), AAV9-BAG5 for DCM (51), AAV9-LAMP2 for Danon disease (81), AAV9-AMPK α2 for transverse aortic constriction (TAC)-induced chronic HF (62), a cell-permeable Tat-Beclin-1 peptide for LPS-induced heart defects (22) and I/R injury (82).

Almost all aspects of cardiac cell function are regulated by a massive series of non-coding RNAs, including miRNAs and lncRNAs (41). Targeting non-coding RNAs of interest provides innovative therapeutic approaches for heart disease treatment by delivering short, antisense oligonucleotides (ASOs). Specific antagomirs against miR-132 safeguard against pressure-overload-induced HF by modulating FoxO3-mediated autophagy (43). As antimiR-132 (also known as CDR132L) shows high therapeutic efficacy in the mouse and pig models of HF (83, 84), this compound has recently entered the clinical trial stage in HF patients (85). LncRNA Chast induces cardiomyocyte hypertrophy and pathological heart remodeling in mice, as Chast impedes cardiomyocyte autophagy by negatively regulating the expression of the autophagy regulator PLEKHM1. Silencing of LncRNA Chast with ASO prevents and improves TAC-induced adverse cardiac remodeling without early signs of toxicity (86). Moreover, silencing of LncRNA 2810403D21Rik/Mirf mitigates cardiac injury and improves heart function in MI mice by promoting miR26a/USP15-mediated autophagy (87).

## Aberrant autophagy in skeletal muscle diseases

Appropriate autophagy is not only essential for cardiac muscle homeostasis and function, but also for maintaining skeletal muscle structure and fitness under basal and stress conditions (88, 89). Autophagy defects lead to various skeletal muscle diseases, as shown in Table 3. Mutations in the core genes related to the autophagy process lead to muscle diseases, as evidenced by the fact that muscle-specific ATG7 deletion results in severe muscle atrophy and an age-dependent decline of force in mice (90) and muscle weakness in human patients (91). Similarly, mice with conditional knockout of ATG5 in skeletal muscle exhibit pronounced muscle wasting, kyphosis, and growth retardation (92). Interestingly, muscle-specific knockout of Vps15 causes the symptoms of autophagic vacuolar myopathy (AVM) with remarkable glycogen accumulation (93). Moreover, skeletal muscle defects are also caused by the mutations of other key autophagic genes, like *Pik3c3* (also known as *Vps34*) (94), *Atg14* (21), *Ulk1*, and *Ulk2* (95). This notion that autophagy is required for muscle fitness is further substantiated by human skeletal muscle diseases with aberrant autophagy and/or accumulation of damaged organelles, such as sarcopenia, muscular dystrophies, and other myopathies (2, 4, 89, 96).

**TABLE 3:** Autophagy in skeletal muscle diseases.

Disease	Target	Model	Main conclusions	References
MDC1A	Lama2	*dy^3*k*^/dy^3*k*^* mice	Increased expression of autophagy-related genes in *dy^3*k*^/dy^3*k*^* mice skeletal muscle	(123)
UCMD	Col6a1	*Col6a1* KO mice	Lower induction of Beclin-1 and Bnip3 and impaired autophagosome formation in KO mice	(117)
LGMDR8	TRIM32	*Trim32* KO mice	TRIM32 is required for autophagy induction by activating ULK1	(111)
LGMDR2	Dysf	LGMDR2 patient	LC3-II, p62, and Bnip3 levels elevate, p62-positive proteins aggregate in patients	(116)
DMD	DYS	*mdx* mice and patients	Autophagy is impaired as AKT is persistently activated	(106)
DM1	MBNL1	Muscle satellite cells	MBNL1 enhances cell proliferation and inhibits autophagy *via* activating mTOR pathway	(136)
DM1	DMPK	DM1 Drosophila and patients	The decrease in muscle area is concomitant with increased apoptosis and autophagy	(137)
DM1	miR-7	DM1 muscle cells	miR-7 restores normal autophagic flux and prevents overexpression of muscle-atrophy-related genes	(138)
Danon	LAMP2	Danon patients	Accumulation and altered localization of VPS15 but TFEB are activated in patients	(119)
Danon	LAMP2	*Lamp2* KO mice	KO mice showed fiber degeneration with an accumulation of vacuoles	(33)
Pompe	GAA	*Atg5/Gaa* dKO mice	Induction of autophagy but impaired autophagosome–lysosome fusion in *Gaa* KO mice	(92)
hIBM	VCP	IBM myoblasts	VCP is essential for maturation of ubiquitin-containing autophagosomes	(130)
hIBM	VCP	*Vcp* KO Drosophila	VCP mutant disrupts tubular lysosomes and impairs autophagosome-lysosome fusion	(131)
hIBM	VCP	*Vcp*-cKO mice	Damaged lysosomes are accumulated in skeletal muscle and persistent TFEB activation in cKO mice	(132)
RVM	p62	RVM patients	Patients have late-onset distal muscle weakness, myopathic features and rimmed vacuoles	(133)
XMEA	VMA21	XMEA patients	VMA21 deficient can raise lysosomal pH which reduces lysosomal degradative ability and activate compensatory autophagy	(135)
Atrophy	ATG7	*Atg7*-cKO mice	Profound muscle atrophy and age-dependent decrease in force, accumulation of abnormal mitochondria in cKO mice	(90)
Myopathy	ATG7	*Atg7* mutation patients	Mild myopathic changes and no vacuoles or internalized nuclei in patients	(91)
Myopathy	ATG5	Atg5-cKO mice	Pronounced muscle wasting, profound kyphosis, and growth retardation in KO mice	(92)
AVM	Vps15	*Vps15* KO mice	Elevated creatine kinase plasma levels, accumulation of autophagosomes, and glycogen in KO mice	(93)
MD	Vps34	*Vps34*-cKO mice	*Vps34*-cKO mice display premature death, dystrophic muscle and aberrant accumulation of membrane-associated proteins	(94)
MD	Sidt2	*Sidt2*-cKO mice	*Sidt2*-cKO mice display muscle weakness and mildly elevated CK with accumulation of autolysosomes, adaptor protein p62 and ubiquitinated aggregates	(141)
SP	HPGD	Aged mice	Suppression of 15-PGDH slowed sarcopenia progression through activating autophagy and facilitating mitochondria biosynthesis	(98)
SP	Apelin	Aged mice, aged human	Apelin enhances muscle function by facilitating autophagy, mitochondrial biogenesis, and anti-inflammatory pathways	(99)
SP	Sesn	*Sesn* KO mice	Sestrins 1–3 maintain muscle mass and strength in aging mice through mTORC1 inhibition and autophagy activation	(100)
SP	GSK-3α	*Gsk-3*α KO mice	Marked activation of mTORC1 and suppression of autophagy markers in KO mice	(101)
Atrophy	Fyn	HSA-*Fyn* TG and KO mice	Fyn/STAT3/Vps34 pathway is responsible for fiber-type-specific regulation of macroautophagy and muscle degeneration	(102)
AVM	Atg14	*Atg14*-cKO, *Rb1cc1*-cKO mice	Atg14-cKO and Rb1cc1-cKO mice display features of AVM with ubiquitin^+^ p62^+^ deposits	(21)
hIBM	ULK1/2	*Ulk1/2* cDKO mice	ULK1 and ULK2 localize to stress granules and ULK-mediated phosphorylation of VCP promotes stress granule disassembly	(95)
SP	Mfn2	*Mfn2*-cKO mice	Mfn2 deficiency reduced autophagy and impaired mitochondrial quality, thereby causing the age−related alterations in metabolic homeostasis and sarcopenia	(104)
MM	mtDNA	Deletor mice and MM patients	Activated or halted mitophagy occur in a mosaic manner in adjacent muscle fibers	(127)
Atrophy	LONP1	*Lonp1*-cKO mice	LONP1 deficiency impairs mitochondrial protein turnover and activates autophagy, thereby causing muscle loss	(140)

MDC1A, congenital muscular dystrophy type 1A; UCMD, Ullrich congenital muscular dystrophy; LGMD, limb girdle muscular dystrophy type 2; DMD, Duchenne muscular dystrophy; DYS, dystrophin; DM1, myotonic dystrophy type 1; hIBM, hereditary inclusion body myopathy; RVM, rimmed vacuolar myopathy; XMEA, X-linked myopathy with excessive autophagy; AVM, autophagic vacuolar myopathy; MD, muscular dystrophy; SP, sarcopenia; MM, mitochondrial myopathy; MEF, mouse embryonic fibroblasts; cDKO, conditional double knock-out; mtDNA, mitochondrial DNA; CK, serum creatine kinase.

### Autophagy in sarcopenia

Sarcopenia, which commonly occurs in elders, is a progressive skeletal muscle disorder characterized by the accelerated loss of muscle mass and function closely linked to increased health concerns, including falls, functional decline, frailty, and even mortality (96). The etiology of sarcopenia is associated with multiple factors, including defective autophagy, where a time-dependent decline in autophagy activity causes stemness impairment in muscle satellite stem cells (96, 97). This tenet is further supported by recent findings demonstrating that suppression of the prostaglandin-degrading enzyme 15-hydroxyprostaglandin dehydrogenase (15-PGDH or HPGD) slowed sarcopenia progression partly through activating autophagy (98) and that exerkine apelin reversed sarcopenia partially by triggering autophagy in mice and humans (99). Autophagy contributes to the maintenance of muscle mass and strength mediated by Sestrins 1–3 in aging mice (100). Glycogen synthase kinase-3 alpha (GSK3α) and Tyrosine-protein kinase (Fyn) are also involved in age-related alterations in sarcopenia by modulating autophagy (101, 102). Furthermore, mitophagy impairment has been associated with sarcopenia, as supported by the observation that the impairment of genes related to mitochondrial fusion or fission contributed to age-dependent muscle degeneration (103). For example, age-dependent loss or genetic disruption of Mfn2 in mouse skeletal muscle causes sarcopenia *via* inhibition of mitophagy (104).

### Autophagy in muscular dystrophy

Duchenne muscular dystrophy (DMD) caused by *DMD* gene mutations is the most common childhood form of muscular dystrophy, with approximately 1 in 5,000 male births worldwide (105). *DMD* codes for the dystrophin protein, a cytoskeletal protein that functions in the muscle force transmission and sarcolemmal stability of muscle fibers. Loss of dystrophin leads to progressive muscle weakness and wasting, loss of ambulation, respiratory impairment, cardiomyopathy, and eventual death. A previous study demonstrated that autophagy was defective at late stages of disease progression in *Dmd* mice and DMD patients (106) and that autophagy impairment correlated with the decline in muscle regeneration and the increase in fibrotic tissue deposition in dystrophic muscles by modulating satellite cell activity (107). Autophagy induction is impaired as mTOR is constitutively activated, leading to the downregulation of LC3, Atg12, Bnip3, and Gabarapl1 in *mdx* mice (106). Moreover, PINK1/PRAK2-mediated mitophagy deficits also contribute to dystrophic phenotypes in a *mdx* mouse model (108).

Limb-girdle muscular dystrophies (LGMDs), the fourth most prevalent genetic muscle disease, are a group of genetically heterogeneous disorders characterized by progressive muscle weakness (5). LGMDs have more than 30 subtypes with variable severity and time of onset, and the pathological mechanism of some types has been associated with aberrant autophagy (109). LGMDR8 (110), characterized by impaired muscle regrowth and atrophy, is caused by mutations in the ubiquitin ligase Tripartite motif-containing protein 32 (TRIM32). TRIM32 is required for autophagy induction in response to atrophic stimuli *in vivo* by catalyzing unanchored K63-linked polyubiquitin of ULK1 and promoting the interaction of ULK1 with autophagy/Beclin 1 regulator 1 (AMBRA1) (111). LGMDR9 is an autosomal recessive disorder defined by proximal muscle weakness, calf hypertrophy, hypotonia and elevated CK level. LGMDR9 is caused by mutations in the fukutin-related protein gene (FKRP) encoding a glycosyltransferase involved in α-dystroglycan modification. A recent finding showed that Atg7 and LC3B-II were markedly increased, but p62 and mTOCR1 were decreased in LGMDR9 patients, indicating that autophagy activation has been linked with disease development (112). Conversely, another study found that autophagy was downregulated in patient-specific LGMDR9 iPSC-derived myotubes (113). LGMDR2 caused by *DYSF* mutation is an autosomal recessive disease, characterized by muscle inflammation, fibrosis and progressive weakness in the hip and shoulder area (114, 115). LGMDR2 patients display elevated LC3-II, p62, and Bnip3 levels (116).

Mutations of COL6A1 encoding collagen type VI has been linked to Ullrich congenital muscular dystrophy (UCMD) characterized by early-onset and generalized muscle weakness, and Bethlem myopathy (BM) characterized by proximal muscle weakness and flexion contractures. Autophagy defects are observed in *Col6a1* deficient mice, in which abnormal AKT-mTOR pathway signaling pathway lowers the induction of Beclin-1 and Bnip3 and impairs autophagosome formation in muscle fibers (117). The massive accumulation of autophagosomes can cause autophagic vacuolar myopathies (AVMs) such as Danon disease (DD) and Pompe disease (4, 118). The causative defect of *LAMP2* leads to Danon disease characterized by weakening of myocardial and skeletal muscles (32). Disruption of LAMP2 expression blocks the normal maturation of autophagosomes in *Lamp2*-deficient mice and impairs the fusion of autophagosome with lysosome in *LAMP2*-deficient human induced pluripotent stem cell-derived cardiomyocytes (hiPSC-CMs) (34, 119, 120). Furthermore, the mutations in *GAA* encoding an acid alpha−glucosidase cause Pompe disease characterized by abnormal buildup of glycogen and muscle weakness. The fusion of autophagosome-lysosome is suppressed while autophagy initiation is induced in *GAA* mutant mouse model (92).

Although the abovementioned muscular dystrophies are associated with defective autophagy, excessive autophagy could cause muscular dystrophies (89, 121). Congenital muscular dystrophy type 1A (MDC1A) is caused by mutations in *LAMA2* encoding the laminin α2 chain. MDC1A is characterized by clinically profound muscle hypotonia and progressive muscle weakness accompanied by contractures (122). Excessive autophagy appears to exacerbate the dystrophic pathologies in the *Lama2*-deficient mouse model and MDC1A patient tissues (123), as evidenced by the observation that a autophagy inhibitor 3-methyladenine (3-MA) improves MDC1A (123). However, the detailed relationship between Laminin α2 and autophagy remains elusive due to a lack of autophagic dynamics.

### Autophagy in other myopathies

Mitochondrial myopathies (MM) are clinically and biochemically heterogeneous disorders characterized by ragged red fibers and peripheral and intermyofibrillar accumulations of abnormal mitochondria (124). The skeletal muscle-specific deletion of *Cox15* encoding a Cytochrome C Oxidase Assembly protein, leads to severe myopathy in mice (125). Meanwhile, rapamycin can improve this myopathy by activating TFEB-mediated lysosome biosynthesis and autophagic flux (126). A recent study found that human patients with MM and Deletor mice (127), a model of adult-onset MM with multiple mtDNA deletions, exhibited overtly abnormal mitophagy by activating mTORC1 (128).

Defects in CASA cause myofibrillar myopathies characterized by Z-band disorganization and rimmed vacuoles (2). Under physiological conditions, CASA targets unfold filamin C for timely autophagic degradation. If CASA is defective, misfolded filamin C and other Z-disc proteins accumulate and impair the integrity of the Z-disc, causing myofibrillar machinery dysfunction (2).

Muscle cells from patients with inclusion body myopathy (IBM) build up ubiquitin-positive rimmed vacuoles and non-digested autophagic vacuoles (129, 130). One of the causative genes for hereditary inclusion body myopathy (hIBM) is *VCP* encoding valosin-containing protein (VCP), whose mutation disrupts the maturation of ubiquitin-containing autophagosomes (130) and the dynamic tubular lysosomal network in fruit flies (131), thereby impairing autophagosome-lysosome fusion. Skeletal muscle-specific KO of *Vcp* in adult mice causes necrotic myopathy with accumulating macroautophagic/autophagic proteins, damaged lysosomes, and persistent activation of TEFB-mediated lysosome biosynthesis (132). The dominantly inherited mutations in *SQSTM1* have been linked to rimmed vacuolar myopathy (RVM) by blocking the aggregated and ubiquitinated proteins to the autophagosome for degradation (133) or perturbing the stress granule dynamics (134). X-linked myopathy with excessive autophagy (XMEA), a childhood onset disease characterized by progressive vacuolation and weakness of skeletal muscle, is attributed to the decrease in Vacuolar ATPase Assembly Factor 21 (VMA21), essential for lysosomal degradative ability by assembling the vacuolar ATPase (135). Moreover, the muscle integrity is also fine-tuned by other autophagic modulators, such as muscle blind-like 1 (MBNL1) (136), myotonic dystrophy protein kinase (DMPK) (137), miR-7 (138), inositol polyphosphate 5-phosphatase (INPP5K) (139), ion protease homolog (LONP1) (140) and Sid1 transmembrane family member 2 (Sidt2) (141).

## Targeting autophagy for skeletal muscle disease treatment

Given that defective autophagy contributes to many skeletal muscle diseases, reactivating autophagy may be beneficial in treating these diseases, as shown in Figure 4 and Table 2. Small molecules, gene therapies, and ASO therapies targeting autophagy have been under development for myopathies (142, 143). Rapamycin improves the pathological manifestations caused by *LMNA* mutations (144), ameliorates the pathology of mitochondrial myopathy (126, 128), and mitigates the myopathic phenotype of *Cox15^*sm*/*sm*^* mice (126). Urolithin A, a natural microflora-derived metabolite that activates mitophagy, improves muscle function in worm and mouse models of DMD (145), and in elderly persons (146). SW033291, specifically inhibiting 15-PGDH-mediated PGE2 signaling, rejuvenates aged muscle mass, strength and exercise capacity partly by increasing autophagy (98). Moreover, the beneficial effects are also observed in other intervention approaches targeting autophagy, like an autophagy agonist spermidine or low protein diet for MDC1A (147, 148), and AAV9-Apelin for sarcopenia (99).

## Summary and perspective

In summary, autophagy plays an important role in the pathogenesis of heart and skeletal muscle diseases. The above-mentioned signaling pathways and molecules are far from being exhaustive, which reflects the rapid development of the field and the complexity of the molecular regulation of autophagy but provides a framework to address the potential analogies between cardiac and skeletal muscle diseases. Some regulatory pathways of autophagy are shared by both cardiac and skeletal myocytes. First, the core machinery of autophagy (such as mTORC1 and AMPK) and CASA commonly play crucial roles in both cardiac and skeletal muscles, suggesting that they may be common therapeutic targets for diseases affecting these two tissues (Figure 4). Second, many muscular dystrophies also exhibit cardiomyopathies (Tables 1, 3). Third, although Rab9-mediated alternative mitophagy has been only demonstrated in the involvement of heart diseases until now, it does not rule out the possibility that this signaling pathway may also be involved in skeletal muscle diseases. Understanding autophagy alterations underlying these diseases has accelerated the development of pharmacological and genetic interventions. The introduction of novel animal models, therapeutic strategies and state-of-the-art approaches for autophagy studies will provide further insights into the roles of autophagy in muscles and facilitate the drug development in the future.

Despite of many studies linking autophagy alterations to various striated muscle pathologies, most employed global or conditional KO animal models to examine autophagy in a snapshot way at certain timepoints. These strategies are limited in several aspects. First, certain autophagy alterations may be a compensatory effect in genetic animal models, as organisms have evolved into sophisticated regulatory mechanisms to safeguard against genetic or environmental insults. Second, autophagy-independent functions of some autophagy-related genes may contribute to the outcomes. Third, autophagy is a highly dynamic process whereas a snapshot of autophagy may not reflect the entire picture. Manipulating autophagy-related genes at the adult stage, pharmacological interventions with high specificity as well as autophagic dynamics analysis will address these limitations in the future.

Traditional and novel experimental approaches studying autophagy in other tissues and diseases can be used to study striated muscle disorders. For instance, single-cell RNA sequencing can determine which cell types contribute to diseases, and establish the link between autophagy and cell types. Specific targets against autophagy in certain cell types will be more beneficial to treatment. Multiomics techniques will provide a broader landscape of the impact of autophagy abnormalities in striated muscle disorders. DNA sequencing applied to human biopsies may determine the relationship between the mutations in autophagy-relevant genes and myopathies. Moreover, High-throughput screening strategies based on cutting-edge CRISPR or RNAi will identify the factors involved in autophagy under physiological or pathophysiological settings of striated muscles.

Some traditional interventions including caloric restriction and small chemicals are not specific and may provoke side effects. Encouragingly, gene therapy and ASO are increasingly being explored to treat autophagy defects in genetic heart and skeletal muscle disorders. Moreover, modulating autophagy *via* novel approaches such as mRNA delivery and gene editing may provide increased efficacy and specificity for treating striated muscle diseases. The drug exploration will be profoundly energized *via* the introduction of novel models such as humanized animal models and human iPSC-derived organoids. Moreover, artificial intelligence and protein structure prediction will boost rationally design of drugs targeting autophagy with higher specificity and efficacy.

## Author contributions

HL primarily wrote the manuscript. All authors listed have made a substantial, direct, and intellectual contribution to the work, and approved it for publication.
